# Supplementary material for: A reverse transcriptase ribozyme
Source: eLife. 2017 Sep 26;6:e31153. doi: 10.7554/eLife.31153 (PMC5665644; doi:10.7554/eLife.31153)
Supplement: Supplementary file 1. [file elife-31153-supp1.docx]

**Supplementary file 1. Sequences of RNA and DNA molecules used in this study.**

| **Type** | **Name** | **R or DNA** | **Source** | **Sequence (5´→3´)** |
| --- | --- | --- | --- | --- |
| PCR primer | Fwd | DNA | com | GGACTAATACGACTCACTATTAGTCATTGCCGCAC |
|  | Rev | DNA | com | GTCAGCCATGTGTTG |
| Ribozyme | 24-3 | RNA | ivt | AGUCAUUGCCGCACGAAAGACAAAUCUGCCCUCAGAGCUUGAGAACAUCUUCGGAUGCAGAGGAGGCAGCCUUCGGUGGAACGAUCGUGCCACCGUUCUCAACACGUACCCGAACGAAAAAGACCUGACAAAAAGGCGUUGUUAGACACGCCCAGGUGCCAUACCCAACACAUGGCUGAC |
| Extension primer | P1 | RNA | syn | FAM-biotin-UUGCUACUACACGAC |
|  | P2 | R/DNA | syn | FAM-biotin-r(UUGCUACUACACGA)-dC |
|  | P3 | DNA | syn | FAM-biotin-TTGCTACTACACGAC |
| Authentic product | r15-d8 | R/DNA | syn | FAM-biotin-r(UUGCUACUACACGAC)-d(CGGGGGTG) |
|  | d23 | DNA | syn | FAM-biotin-TTGCTACTACACGACCGGGGGTG |
| Template | T1 | RNA | ivt | rev: TTGCTACTACACGACCGGGGGTGTTTGTCATTGTCTATAGTGAGTCGTATTAGCC tx: GACAAUGACAAACACCCCCGGUCGUGUAGUAGCAA |
|  | T2 | RNA | ivt | rev: TTGCTACTACACGACCGGGGGTGCGGGGGAGTTTTTGTCATTGTCTATAGTGAGTCGTATTAGCC tx: GACAAUGACAAAAACUCCCCCGCACCCCCGGUCGUGUAGUAGCAA |
|  | T3 | RNA | ivt | rev: TTGCTACTACACGACGAGTGGTGAGGCAGAGTTTTTGTCATTGTCTATAGTGAGTCGTATTAGCC tx: GACAAUGACAAAAACUCUGCCUCACCACUCGUCGUGUAGUAGCAA |
|  | T4 | RNA | ivt | rev: TTGCTACTACACGACGCGAGGAGTGTGTGTGTTTTTGTCATTGTCTATAGTGAGTCGTATTAGCC tx: GACAAUGACAAAAACACACACACUCCUCGCGUCGUGUAGUAGCAA |
|  | T5 | RNA | ivt | rev: TTGCTACTACACGACCGGGGGTGCGGGGGAGCGGGGGTGCGGGGGAGTTTTTGTCATTGTCTATAGTGAGTCGTATTAGCC tx: GACAAUGACAAAAACUCCCCCGCACCCCCGCUCCCCCGCACCCCCGGUCGUGUAGUAGCAA |
|  | T6 | RNA | ivt | rev: TTGCTACTACACGACGTGTGGAGTGCGTGTGTTTTTGTCATTGTCTATAGTGAGTCGTATTAGCC tx: GACAAUGACAAAAACACACGCACUCCACACGUCGUGUAGUAGCAA |
|  | dT4 | DNA | com | GACAATGACAAAAACACACACACTCCTCGCGTCGTGTAGTAGCAA |
|  | dT6 | DNA | com | GACAATGACAAAAACACACGCACTCCACACGTCGTGTAGTAGCAA |

The molecules were synthesized in-house (syn), purchased from IDT (com), or prepared by in vitro transcription (ivt). The PCR primers were used to amplify a portion of plasmid DNA encoding the 24-3 ribozyme. The T7 RNA polymerase promoter sequence is underlined. The forward primer for preparing DNAs encoding templates T1–T6 had the sequence 5´-GGCTAATACGACTCACTATA-3´. Sequences in red indicate the tag used on the ribozyme and templates to improve processivity. Sequences in blue indicate the primer binding site. FAM, 6-fluorescein label.
